# Índice Imunoinflamatório Sistêmico como Determinante de Carga Aterosclerótica e Pacientes de Alto Risco com Síndromes Coronarianas Agudas

**DOI:** 10.36660/abc.20210416

**Published:** 2022-06-23

**Authors:** Demet Ozkaramanli Gur, Muhammet Mucip Efe, Seref Alpsoy, Aydın Akyüz, Nurullah Uslu, Aliye Çelikkol, Ozcan Gur

**Affiliations:** 1 Namik Kemal University Faculty of Medicine Department of Cardiology Tekirdag Turquia Namik Kemal University,Faculty of Medicine, Department of Cardiology, Tekirdag – Turquia; 2 Namik Kemal University Faculty of Medicine Biochemistry Department Tekirdag Turquia Namik Kemal University,Faculty of Medicine, Biochemistry Department, Tekirdag – Turquia; 3 Namik Kemal University Faculty of Medicine Department of Cardiovascular Surgery Tekirdag Turquia Namik Kemal University,Faculty of Medicine, Department of Cardiovascular Surgery, Tekirdag – Turquia

**Keywords:** Inflamação, Síndrome Coronariana Aguda, Doença da Artéria Coronariana

## Abstract

**Fundamento:**

O índice imunoinflamatório sistêmico (IIS), derivado das contagens de neutrófilos, plaquetas e linfócitos, representa o equilíbrio homeostático entre os estados inflamatório, imune e trombótico. O IIS é superior a índices como a relação neutrófilos-linfócitos no prognóstico de várias malignidades, além de ser um melhor preditor de futuros eventos cardíacos que os fatores de risco tradicionais após a intervenção coronariana.

**Objetivos:**

Este estudo objetivou avaliar a relação do IIS com a carga aterosclerótica e complicações hospitalares em pacientes com síndrome coronariana aguda.

**Métodos:**

Desfechos clínicos, como extensão do dano miocárdico, carga aterosclerótica, sangramento, insuficiência renal aguda, duração da internação e mortalidade hospitalar, foram avaliados em uma coorte retrospectiva de 309 pacientes consecutivos com síndrome coronariana aguda. O IIS foi calculado como (plaqueta x neutrófilos)/contagem de linfócitos na admissão. A população estudada foi categorizada em tercis de IIS. Valores de p<0,05 foram considerados estatisticamente significativos.

**Resultados:**

Os maiores valores de IIS foram encontrados em pacientes com infarto do miocárdio com supradesnivelamento do segmento ST (641,4 com angina pectoris instável, 843,0 com infarto do miocárdio sem supradesnivelamento do segmento ST e 996,0 com infarto do miocárdio com supradesnivelamento do segmento ST; p=0,004). Concentração máxima de troponina (0,94 versus 1,26 versus 3; p<0,001), número de vasos doentes (1 versus 2 versus 2; p<0,001), escore SYNTAX ( *The SYNergy between percutaneous coronary intervention with TAXus and cardiac surgery* — sinergia entre intervenção coronária percutânea com taxus e cirurgia cardíaca) (9 versus 14 versus 17,5; p<0,001) e duração da internação (2 versus 2 versus 3; p<0,001) também aumentaram de acordo com o tercil de IIS (tercil 1 versus tercil 2 versus tercil 3). O IIS foi um preditor independente de escore SYNTAX (ß: 0,232 [0,001 a 0,003]; p<0,001), extensão do dano miocárdico (ß: 0,152 [0 a 0,001]; p=0,005) e duração da internação (ß: 0,168 [0,0 a 0,001]; p=0,003).

**Conclusões:**

Este estudo demonstrou que o IIS, um índice hematológico simples, é um marcador melhor de carga aterosclerótica e internação mais longa do que fatores de risco bem conhecidos em pacientes com síndrome coronariana aguda de alto risco.

## Introdução

A aterosclerose é caraterizada por uma inflamação crônica de baixo grau, interrompida por períodos de exacerbações agudas. Esses picos da resposta inflamatória aceleram o processo da doença e se manifestam clinicamente como síndromes coronarianas agudas (SCA).^1 , 2^ A SCA é comum em pacientes com doença arterial coronariana (DAC), resultando em altas taxas de mortalidade e morbidade.^3^ A estratificação precisa do risco no curso inicial da doença, portanto, é de suma importância.

Há vários marcadores inflamatórios, como a proteína C reativa (PCR), o fator de necrose tumoral-α e várias interleucinas, que estão associados a um desfecho pior no contexto da SCA.^4 , 5^ Índices hematológicos simples, como a relação neutrófilos-linfócitos (RNL) e a relação plaquetas-linfócitos (RPL), também são indicadores úteis de inflamação e fatores prognósticos promissores para a doença cardiovascular.^6 , 7^

Na SCA, a ativação descontrolada da imunidade inata e adaptativa converge com a ativação plaquetária, resultando na formação de trombos. O índice imunoinflamatório sistêmico (IIS), derivado das contagens de plaquetas, neutrófilos e linfócitos, combina os principais atores dessas vias fisiopatológicas para representar o comprometimento do equilíbrio. Foi descrito pela primeira vez como uma ferramenta prognóstica no carcinoma hepatocelular,^8^ seguido por outros tumores sólidos, como os dos cânceres colorretal, esofágico e cervical.^9^ Demonstrou-se que o IIS é um melhor preditor de sobrevida do que outros índices hematológicos, como a RNL ou a RPL em malignidades.^9^ Recentemente, Yang et al.^10^ revelaram ainda que o IIS é um melhor preditor de eventos cardiovasculares maiores do que fatores de risco cardiovascular bem conhecidos em pacientes submetidos à intervenção coronariana percutânea (ICP). Este estudo, portanto, teve como objetivo explorar a associação do IIS na admissão com a carga aterosclerótica e desfechos clínicos iniciais para identificar pacientes de alto risco com SCA.

## Métodos

**Figura 1:**
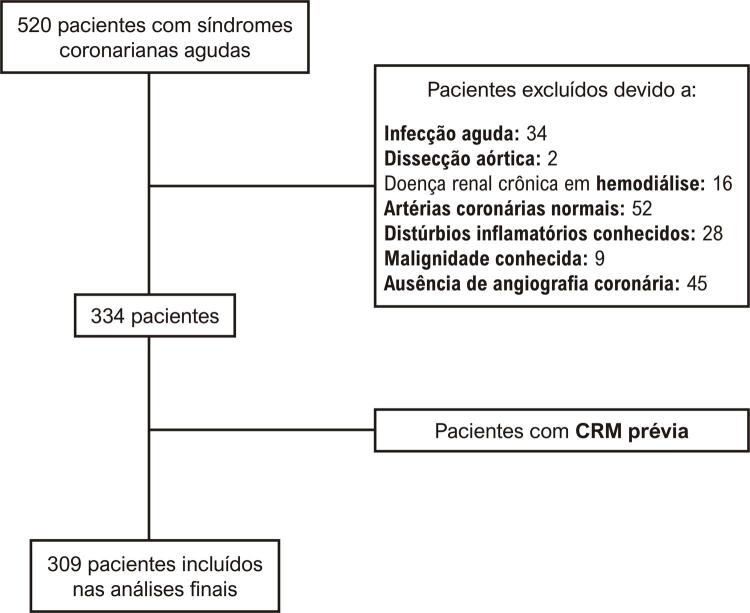
Fluxograma da seleção de pacientes. CRM: cirurgia de revascularização do miocárdio.

Após a aprovação do comitê de ética local (2019/28/02/12), foram avaliados retrospectivamente pacientes com SCA que visitaram o pronto-socorro e foram tratados com angiografia coronariana entre janeiro de 2018 e janeiro de 2019. Foram identificados 520 pacientes consecutivos diagnosticados com SCA — ou seja, angina pectoris instável (API), infarto agudo do miocárdio sem supradesnivelamento do segmento ST (IAMSSST) e infarto do miocárdio com supradesnivelamento do segmento ST (IAMCSST) — com base em suas caraterísticas eletrocardiográficas, clínicas e laboratoriais.^11 , 12^ Pacientes com altas concentrações de troponina devido a uma doença diferente da SCA, com doença inflamatória/infeciosa conhecida, em hemodiálise e com diagnóstico de infarto do miocárdio (IM) com artérias coronárias normais, assim como aqueles que não foram submetidos à angiografia coronariana durante a internação inicial não foram incluídos no estudo ( Figura 1 ). A partir dos prontuários médicos de 334 pacientes elegíveis, foram coletadas caraterísticas demográficas, como idade, gênero e presença de fatores de risco cardiovascular, incluindo hipertensão, dislipidemia e diabetes mellitus (DM). Pacientes previamente submetidos à cirurgia de revascularização do miocárdio (CRM) não foram incluídos nas análises, pois a associação do IIS com a carga aterosclerótica não pôde ser estratificada nessa população específica. Parâmetros laboratoriais, como hemoglobina, concentração de ureia e creatinina, bem como contagens de neutrófilos, plaquetas e linfócitos, foram determinados na admissão na unidade de emergência. Os desfechos clínicos identificados para avaliação foram carga aterosclerótica, extensão do dano miocárdico, sangramento, insuficiência renal aguda, duração da internação e mortalidade hospitalar.

Para determinar a carga aterosclerótica, angiogramas coronarianos foram avaliados por dois cardiologistas, cegados aos grupos de estudo, que analisaram o número de vasos doentes e os escores SYNTAX ( *The SYNergy between percutaneous coronary intervention with TAXus and cardiac surgery* — sinergia entre intervenção coronária percutânea com taxus e cirurgia cardíaca). Qualquer artéria coronária epicárdica com 50% ou mais de estenose foi considerada um vaso doente. O escore SYNTAX foi calculado conforme descrito anteriormente.^13^ O máximo de troponina I ultrassensível (hs-cTnI) representou a extensão do dano miocárdico. Sangramento foi definido como uma queda de hemoglobina (Hb) de 3 g/dL ou mais durante a internação. Níveis de creatinina (Cr) foram obtidos durante o período de internação para calcular o aumento em relação ao nível de referência de Cr, sendo que um aumento de >0,3 mg/dL ou 1,5 vez o valor de referência indicou a ocorrência de insuficiência renal aguda. O IIS foi calculado como (plaquetas x neutrófilos/linfócitos), como descrito e estudado anteriormente.^8^ A população do estudo foi estratificada em três grupos com base nos níveis de IIS.

### Análise estatística

As análises estatísticas foram realizadas no programa IBM^®^ SPSS^®^ Statistics para Mac, versão 20 (IBM Corp., Armonk, Nova York). Variáveis contínuas foram apresentadas como média±desvio padrão (DP) ou mediana (intervalo interquartil); variáveis categóricas foram descritas como número e porcentagens. A distribuição normal foi testada nas variáveis contínuas pelo teste de Kolmogorov-Smirnov. Os tercis de IIS foram comparados pela análise de variância (ANOVA) de uma via em variáveis com distribuição normal e pelo teste de Kruskal-Wallis em variáveis sem distribuição normal. O teste de Dunn foi utilizado em comparações pareadas não paramétricas se desvios significativos fossem observados no teste de Kruskal-Wallis. No caso de desvios significativos revelados pela ANOVA, a análise post hoc foi planejada para ser realizada utilizando o teste de Tukey ou Tamhane, dependendo da homogeneidade das variâncias. Nenhuma análise post hoc foi feita para variáveis derivadas do IIS. Variáveis categóricas foram comparadas pelo teste qui-quadrado. Utilizou-se o valor de p=0,017, ajustado pelo método de Bonferroni, nas comparações pareadas de variáveis categóricas.

A correlação dos índices hematológicos IIS, RNL e RPL com o escore SYNTAX em relação ao tipo de SCA foi testada pelo coeficiente de correlação de postos de Spearman. Com a definição de cada desfecho clínico como variável dependente, foram realizadas análises de regressão linear e logística. Variáveis com valor de p≤0,2 nas comparações univariadas foram incluídas no modelo multivariado usando o método *stepwise* para determinar se o IIS foi preditor desse desfecho clínico específico. Todas as suposições necessárias para o uso da análise de regressão linear foram verificadas antes da interpretação dos resultados. Os resultados são apresentados com intervalo de confiança de 95% dentro de [colchetes]. Variáveis de referência como idade, gênero, fatores de risco cardiovascular, Cr, PCR e IIS apresentadas na  Tabela 1  foram incluídas nas análises univariadas para determinar possíveis preditores, mas apenas aquelas com valor de p≤0,2 foram incluídas no modelo multivariado. Valores de p<0,05 foram considerados estatisticamente significativos.

**Tabela 1:** Características de referência e comparação destas características com base nos tercis do índice imunoinflamatório sistêmico

Variável	Total n=309	Tercil 1 do IIS n=103	Tercil 2 do IIS n=103	Tercil 3 do IIS n=103	1 versus 2 Valor de p	2 versus 3 Valor de p	1 versus 3 Valor de p
Idade, anos	64,5±12,1	62,8±11,8	65,1±13,1	65,5±11,2	0,500	1,00	0,306
Gênero feminino, n (%)	82 (26,5)	25 (24,3)	32 (31,1)	25 (24,3)	0,350	0,350	1,00
API, n (%)	53 (17,2)	27 (26,2)	17 (16,5)	9 (8,7)	0,125	0,141	0,002
IAMSSST, n (%)	113 (36,6)	36 (35)	46 (44,7)	31 (30,1)	0,200	0,043	0,552
IAMCSST, n (%)	143 (46,3)	40 (38,8)	40 (38,8)	63 (61,2)	1,00	0,002	0,002
Diabetes mellitus, n (%)	123 (39,8)	36 (35)	40 (38,8)	47 (45,6)	0,665	0,397	0,155
Hipertensão, n (%)	117 (37,9)	43 (41,7)	33 (32)	41 (39,8)	0,194	0,309	0,887
Dislipidemia, n (%)	125 (40,5)	42 (40,8)	36 (35)	47 (45,6)	0,473	0,155	0,574
Hemoglobina, g/dL	13,5±1	13,8±1,8	13,2±1,8	13,3±1,8	0,047	1,00	0,075
Leucócitos, 10^3^/µL	9,9±2,7	9,1±2,7	9,5±2,3	11,4±2,5	0,571	<0,001	<0,001
Contagem de neutrófilos, 10^3^/µL	6,8 (3,54)	5,1 (2,37)	6,7 (2,5)	8,8 (3,03)	<0,001	<0,001	<0,001
Contagem de linfócitos, 10^3^/µL	1,84 (1,1)	2,3 (1,12)	1,8 (0,89)	1,3 (0,75)	<0,001	<0,001	<0,001
Contagem de monócitos, 10^3^/µL	0,63 (0,36)	0,7 (0,36)	0,6 (0,31)	0,6 (0,39)	0,163	0,893	0,341
Contagem de plaquetas, 10^3^/µL	238 (96)	207 (75,75)	241 (75,00)	278 (116,75)	0,001	<0,001	<0,001
Proteína C reativa, mg/L	4,55 (8,63)	3,8 (8,1)	4,7 (8,1)	5,4 (10,8)	0,651	0,262	0,271
Ureia, mg/dL	34 (15)	32 (13)	33,5 (15)	36 (17,1)	0,469	0,082	0,038
Creatinina, mg/dL	0,93 (0,32)	0,93 (0,28)	0,91 (0,33)	0,94 (0,38)	0,984	0,694	0,854
IIS	835 (860,09)	462 (194,51)	833 (252,9)	2055 (935,7)	<0,001	<0,001	<0,001

*Os valores de p representam as comparações pareadas dos tercis do índice imunoinflamatório sistêmico dos três grupos de pacientes sem análise post hoc. É importante notar que, de acordo com a correção de Bonferroni, o nível de significância para o valor de p é 0,017 nesta tabela. API: angina pectoris instável; IAMSSST: infarto agudo do miocárdio sem supradesnivelamento do segmento ST; IAMCSST: infarto agudo do miocárdio com supradesnivelamento do segmento ST; IIS: índice imunoinflamatório sistêmico.

## Resultados

**Figura 2:**
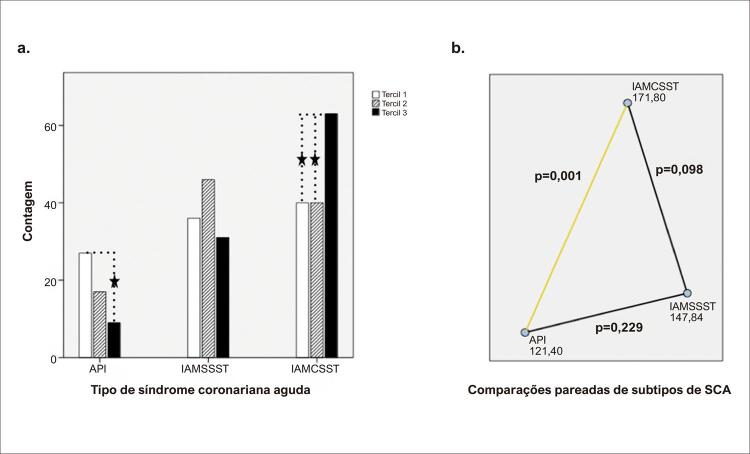
(a) Comparação dos grupos do índice imunoinflamatório sistêmico em relação ao tipo de síndrome coronariana aguda; (b) comparação do tipo de síndrome coronariana aguda dentro do espectro do índice imunoinflamatório sistêmico. API: angina pectoris instável; IAMSSST: infarto agudo do miocárdio sem supradesnivelamento do segmento ST; IAMCSST: infarto agudo do miocárdio com supradesnivelamento do segmento ST; SCA: síndrome coronariana aguda.

As caraterísticas de referência da população estudada e a comparação dessas caraterísticas em relação aos tercis de IIS são apresentadas na Tabela 1 . Não houve diferença entre os grupos investigados quanto a fatores de risco cardiovascular, como idade, gênero, DM, hipertensão e dislipidemia. Quando os grupos de IIS foram comparados com relação ao tipo de SCA, pacientes com API tinham maior probabilidade de estarem no tercil mais baixo de IIS, enquanto os com IAMCSST apresentaram maior probabilidade de terem ISS mais elevado. Pacientes com API tinham maior probabilidade de estarem no tercil mais baixo de IIS, enquanto os com IAMCSST apresentaram maior probabilidade de terem IIS mais elevado ( Figura 2a ). Para identificar a associação entre apresentação clínica e o IIS, o tipo de SCA foi avaliado dentro do espectro do IIS, indicando que o IIS diferiu significativamente em relação ao tipo de SCA. As comparações pareadas revelaram que o IIS aumentou gradualmente da API para o IAMCSST, sendo a diferença entre eles estatisticamente significante ( Figura 2b ). A concentração de hemoglobina, ureia, creatinina e os níveis de PCR foram comparáveis nos subgrupos de IIS.

**Figura 3:**
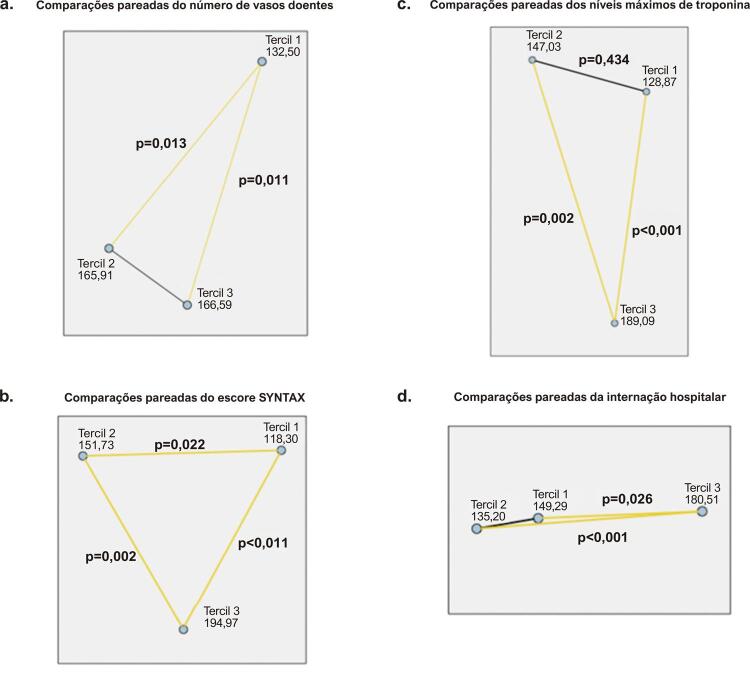
Comparação pareada dos tercis do índice imunoinflamatório sistêmico em relação a (a) número de vasos doentes; (b) escore SYNTAX; (c) níveis máximos de troponina; (d) internação hospitalar. SYNTAX: The SYNergy between percutaneous coronary intervention with TAXus and cardiac surgery (sinergia entre intervenção coronária percutânea com taxus e cirurgia cardíaca).

A Tabela 2 resume os desfechos clínicos nos grupos de IIS. A carga e a complexidade da doença arterial coronariana refletiram-se no número de vasos doentes e no escore SYNTAX de cada paciente, ambos com diferença significativa entre os grupos de IIS. Houve um número significativamente menor de vasos doentes no tercil mais baixo de IIS em relação a outros grupos de IIS ( Figura 3a ). Além disso, o escore SYNTAX aumentou substancialmente à medida que o tercil de IIS cresceu ( Figura 3b ). O nível máximo de troponina — marcador de dano miocárdico — aumentou de acordo com o crescimento do tercil de IIS, sendo as diferenças entre os tercis 1 e 3 e entre os tercis 2 e 3 estatisticamente significativas ( Figura 3c ). A internação hospitalar foi mais longa em pacientes com IIS mais alto em comparação com os outros dois grupos de IIS ( Figura 3d ). Não houve diferença significativa entre os grupos de IIS em termos de sangramento, insuficiência renal aguda ou mortalidade hospitalar.

**Tabela 2:** Desfechos clínicos em relação aos tercis do índice imunoinflamatório sistêmico

Desfecho	Tercil 1 do IIS n=103	Tercil 2 do IIS n=103	Tercil 3 do IIS n=103	1 versus 2 Valor de p	2 versus 3 Valor de p	1 versus 3 Valor de p
Número de vasos doentes	1 (1)	2 (2)	2 (2)	0,013	1,00	0,011
Carga aterosclerótica ou Escore SYNTAX	9 (11)	14 (11,5)	17,5 (11)	0,022	0,002	<0,001
Extensão do dano miocárdico ou troponina máxima, ng/L	0,94 (2,06)	1,26 (3,61)	3 (4,02)	0,434	0,002	<0,001
Internação, dias	2 (1)	2 (1)	3 (3)	0,709	<0,001	0,026
Sangramento, n (%)	27 (26,2)	22 (21,4)	32 (31,1)	0,257*	0,157*	0,538*
Insuficiência renal aguda, n (%)	18 (17,5)	17 (16,5)	22 (21,4)	1,00*	0,477*	0,598*
Mortalidade hospitalar, n (%)	5 (4,9)	8 (7,8)	9 (8,7)	0,568*	1,00*	0,407*

*É importante notar que, de acordo com a correção de Bonferroni, o nível de significância para o valor de p é 0,017 para variáveis categóricas. IIS: índice imunoinflamatório sistêmico; SYNTAX: The SYNergy between percutaneous coronary intervention with TAXus and cardiac surgery (sinergia entre intervenção coronária percutânea com taxus e cirurgia cardíaca).

Foram avaliadas as correlações dos índices hematológicos IIS, RNL e RPL com o escore SYNTAX quanto ao tipo de SCA ( Tabela 3 ). Em pacientes com API, os índices RNL e RPL não se correlacionaram com o escore SYNTAX; já o IIS apresentou correção positiva com o escore SYNTAX. Em pacientes com IAMSSST, tanto IIS, quanto RNL e RPL foram positivamente correlacionados com o escore SYNTAX. Da mesma forma, todos os três índices apresentaram correlação positiva com o escore SYNTAX em pacientes com IAMCSST.

**Tabela 3:** Correlação do escore SYNTAX com o índice imunoinflamatório sistêmico, relação neutrófilos-linfócitos e relação plaquetas-linfócitos em relação ao tipo de síndrome coronariana aguda

		IIS			RNL		RPL	
		r_s_	p		r_s_	p	r_s_	p
API	SYNTAX	0,300	0,031		0,266	0,054	0,145	0,299
IAMSSST	SYNTAX	0,345	<0,001		0,236	0,011	0,183	0,045
IAMCSST	SYNTAX	0,471		<0,001	0,456	<0,001	0,387	<0,001

*IIS: índice imunoinflamatório sistêmico; RNL: relação neutrófilos-linfócitos; RPL: relação plaquetas-linfócitos; API: angina pectoris instável; IAMSSST: infarto agudo do miocárdio sem supradesnivelamento do segmento ST; IAMCSST: infarto agudo do miocárdio com supradesnivelamento do segmento ST; SYNTAX: The SYNergy between percutaneous coronary intervention with TAXus and cardiac surgery (sinergia entre intervenção coronária percutânea com taxus e cirurgia cardíaca).*

Os desfechos clínicos testados pelas análises de regressão linear multivariadas foram a carga aterosclerótica (representada pelo escore SYNTAX), a extensão do dano miocárdico (concentração máxima de troponina) e a duração da internação. A regressão linear revelou que o IIS (ß: 0,232 [0,001 a 0,003]; p<0,001), a idade (ß: 0,156 [0,019 a 0,165]; p=0,014) e a DM (ß: 0,165 [0,935 a 4,42]; p=0,003) foram preditores significativos do escore SYNTAX. Os preditores independentes da extensão do dano miocárdico foram o IIS (ß: 0,152 [0 a 0,001]; p=0,005); o gênero feminino (ß: -0,147 [-1,801 a -0,271]; p=0,008) e a DM (ß: 0,142 [0,197 a 1,557]; p=0,012). De forma semelhante, os preditores independentes de duração da internação foram o IIS (ß: 0,168 [0,0 a 0,001]; p=0,003) e a DM (ß: 0,124 [0,095 a 1,74]; p=0,029).

Os desfechos clínicos testados pelas análises de regressão logística foram sangramento e mortalidade hospitalar. A regressão logística binária indicou que um preditor independente de sangramento foi o valor de referência da hemoglobina ( *odds ratio* — OR: 1,29 [1,09 a 1,52]; p=0,002). Para mortalidade hospitalar, somente a idade (OR: 1,09 [1,035 a 1,155]; p=0,001) e a Cr de referência (OR: 4,6 [1,137 a 18,787]; p=0,032) foram preditores. O IIS não foi preditor de nenhum dos desfechos.

## Discussão

Nessa coorte retrospectiva de pacientes com SCA, demonstrou-se que um simples índice hematológico derivado da contagem de neutrófilos, linfócitos e plaquetas na admissão pode ser utilizado para extrapolar a carga aterosclerótica, a extensão do dano miocárdico e a duração da internação hospitalar, independentemente dos fatores de risco cardiovascular tradicionais e marcadores inflamatórios, como a PCR. A correlação do IIS com o escore SYNTAX persistiu em pacientes com SCA, independentemente da presença ou ausência de necrose. Esse achado sugere que o IIS pode potencialmente ser usado para identificar indivíduos de alto risco com SCA já na admissão.

A mortalidade e a morbidade em eventos cardiovasculares são multifatoriais e resultam de uma confluência de diferentes vias fisiopatológicas, nas quais a inflamação desempenha um papel central.^1^ Embora a inflamação sistêmica grave seja um indicador estabelecido de mortalidade na SCA, nenhum biomarcador inflamatório único é capaz de orientar o tratamento do risco cardiovascular.^14^ Mesmo a PCR, cujo papel na inflamação e na aterosclerose está bem estabelecido, só consegue predizer eventos cardiovasculares moderadamente.^15^ Vários índices hematológicos, como a RNL e a RPL, têm sido propostos para representar a resposta inflamatória precoce na SCA com significância prognóstica.^16^ No entanto, a inflamação é um processo contínuo e evidências revelam que um grande número de citocinas circulantes diminuem após um evento agudo, com persistência de altas concentrações de algumas citocinas, como a interleucina-6 no início do período pós-IM.^17^

Entre os subtipos de leucócitos, os neutrófilos são os principais elementos da primeira linha de defesa não específica. A contagem de neutrófilos é um fator prognóstico bem definido na doença cardiovascular, particularmente em pacientes com SCA.^18 , 19^ Os linfócitos, por outro lado, fazem parte da imunidade adaptativa, que alivia a inflamação por meio dos subtipos B_1_, T-auxiliar_2_ e T-regulador.^1 , 20^ A contagem de linfócitos diminui secundária aos hormônios de estresse agudo após o IM.^21^ Estudos clínicos também estabeleceram a associação da baixa contagem de linfócitos com o aumento da mortalidade hospitalar.^21^ Assim, uma RNL alta está relacionada a piores desfechos clínicos, tanto em pacientes com SCA quanto em portadores de DAC estável submetidos à ICP.^6 , 7^ A RNL também mostrou estar associada à gravidade e à complexidade da DAC, representada pelo escore SYNTAX.^6^

Com base em evidências que demonstram uma interação leucócitos-plaquetas próxima na trombose desencadeada por inflamação, Choi et al.^22^ combinaram a RNL com o volume plaquetário médio e revelaram que a adição de um índice relacionado a plaquetas melhora a predição de futuros eventos cardíacos, especialmente em pacientes com SCA. Çiçek et al.^7^ também demonstraram que uma combinação de RNL e RPL aumentou o poder de predizer um prognóstico pior a curto e longo prazo comparado ao uso isolado destes marcadores em pacientes submetidos à ICP primária. Neste estudo, especulou-se que um índice que combinasse RNL e RPL representaria melhor o estado inflamatório-trombótico dos pacientes no período peri-IM.

Há poucos dados sobre o valor do IIS em doenças cardiovasculares. Yang et al.^10^ avaliaram o papel do IIS em pacientes submetidos à ICP e identificaram que, em um seguimento de 54,6±35,1 meses, o IIS foi um melhor preditor de eventos cardíacos maiores, como morte cardiovascular, IM não fatal e acidente vascular cerebral não fatal, do que fatores de risco cardiovascular tradicionais. Eles sugeriram que a inflamação quantificada pelo IIS representou os piores desfechos clínicos. No entanto, eles não reportaram a associação do IIS com a complexidade da DAC. Pela primeira vez na literatura, demonstrou-se que o IIS apresenta uma associação independente com o escore SYNTAX, independentemente da PCR e de outros fatores de risco cardiovascular conhecidos. Este achado também explica os resultados de Yang et al.,^10^ uma vez que pacientes com IIS e escores SYNTAX mais elevados são mais propensos a eventos cardiovasculares futuros.

Neste trabalho, foi interessante encontrar uma correlação positiva entre o IIS e o escore SYNTAX em todos os subtipos de SCA. Dentre os três índices hematológicos, ou seja, RNL, RPL e IIS, somente o IIS foi correlacionado com o escore SYNTAX em pacientes com API sem necrose miocárdica grave. Estudos sobre SCA mostrando a relação da RNL e da RPL com a carga aterosclerótica foram conduzidos principalmente com pacientes com IAMSSST ou IAMCSST com necrose miocárdica grave e concentrações de troponina significativamente elevadas.^23 , 24^ Além disso, o IIS foi profundamente influenciado pelo tipo de SCA, com IIS substancialmente mais elevado em pacientes com IAMCSST e mais baixo em pacientes com API. Esses achados sugerem que, ao contrário da simples percepção que considera o IIS como resultado do pico inflamatório por volta da ocorrência da SCA, o IIS reflete o processo aterosclerótico interligado de referência, propenso a complicações.

O IIS foi relacionado com a extensão do dano miocárdico e essa relação provavelmente se deu por meio do tipo de SCA, com valores mais elevados de IIS em pacientes com IAMCSST. Este achado está de acordo com estudos anteriores, cujos resultados demonstraram que, na ausência de necrose, a correlação da contagem de leucócitos com a mortalidade diminuiu.^25 , 26^ Núñez et al.^26^ encontraram uma associação mais fraca entre leucócitos e o IAMSSST comparado ao IAMSCSST e especularam que quanto maior a extensão da necrose, maior a resposta dos leucócitos. Os resultados da presente pesquisa sugerem que o IIS, que integra a contagem plaquetária às contagens de leucócitos, pode definir melhor a inflamação residual contínua do que outros índices hematológicos, mesmo na ausência de necrose.

Neste estudo, o IIS foi um preditor de duração da internação hospitalar em pacientes com SCA. O aumento dos valores de IIS representa pacientes de alto risco com escore SYNTAX elevado e dano miocárdico grave, explicando o atendimento hospitalar mais longo. Esta investigação não encontrou associação entre IIS e mortalidade hospitalar, o que pode estar atribuído ao tratamento precoce com intervenção percutânea primária e o tempo de internação relativamente curto.

### Limitações do estudo

Este estudo apresenta diversas limitações relativas à natureza retrospectiva de seu delineamento e à falta de dados de seguimento. A seleção da amostra foi elaborada, pois tentou-se excluir todos os pacientes com infeção ativa ou condição inflamatória, diminuindo assim o número de participantes. Ademais, não foram incluídos pacientes com CRM prévia, já que a carga aterosclerótica não poderia ser determinada pelo número de vasos doentes ou pelo escore SYNTAX nestes indivíduos. Outra limitação do presente estudo foi não incluir nas análises a medicação de referência da população estudada.

## Conclusões

O equilíbrio imune, inflamatório e trombótico é fundamental na patogênese da SCA. Dada sua associação distinta com o escore SYNTAX, a extensão do dano miocárdico e a duração da internação, o IIS pode ser potencialmente usado para identificar pacientes de alto risco por meio de uma abordagem prontamente disponível e de baixo custo.
