# Supplementary material for: Índice Imunoinflamatório Sistêmico como Determinante de Carga Aterosclerótica e Pacientes de Alto Risco com Síndromes Coronarianas Agudas
Source: Arq Bras Cardiol. 2022 Jun 23;119(3):382–90. [Article in Portuguese] doi: 10.36660/abc.20210416 (PMC9438541; doi:10.36660/abc.20210416)
Supplement: Translation

# Translation: Systemic Immune-Inflammatory Index as a Determinant of Atherosclerotic Burden and High-Risk Patients with Acute Coronary Syndromes

**Authors:** Demet Ozkaramanli Gur, Muhammet Mucip Efe, Seref Alpsoy, Aydın Akyüz, Nurullah Uslu, Aliye Çelikkol, Ozcan Gur

**Affiliations:** 1 Turkey Namik Kemal University,Faculty of Medicine, Department of Cardiology, Tekirdag – Turkey; 2 Turkey Namik Kemal University, Faculty of Medicine, Biochemistry Department, Tekirdag – Turkey; 3 Turkey Namik Kemal University, Faculty of Medicine, Department of Cardiovascular Surgery, Tekirdag – Turkey

**Keywords:** Inflammation, Acute Coronary Syndrome, Coronary Artery Disease

## Abstract

**Background:**

Systemic immune-inflammatory index (SII), which is derived from neutrophil, platelet and lymphocyte counts, represents the homeostatic balance among inflammatory, immune and thrombotic status. The systemic immune-inflammatory index is superior to indices such as neutrophil-lymphocyte ratio in predicting prognosis in various malignancies, while it is shown to predict future cardiac events better than traditional risk factors after coronary intervention.

**Objectives:**

Herein, we aimed to evaluate the relationship of the systemic immune-inflammatory index with atherosclerotic burden and in-hospital complications in acute coronary syndrome patients.

**Methods:**

The clinical outcomes, such as extent of myocardial damage, atherosclerotic burden, bleeding, acute kidney injury, duration of hospital stay and in-hospital mortality, were evaluated in a retrospective cohort of 309 consecutive acute coronary syndrome patients. The systemic immune-inflammatory index was calculated as (Platelet X Neutrophil)/Lymphocyte count on admission. Study population was categorized into tertiles with regard to systemic immune-inflammatory index. A p value of <0.05 was considered statistically significant.

**Results:**

The highest systemic immune-inflammatory index values were within ST elevation myocardial infarction patients (641.4 in unstable angina pectoris, 843.0 in non-ST elevation myocardial infarction patients and 996.0 in ST elevation myocardial infarction patients; p=0.004). Maximal troponin concentration (0.94 vs. 1.26 vs. 3; p<0.001), number of diseased vessels (1 vs. 2 vs. 2; p<0.001), the SYNTAX (synergy between percutaneous coronary intervention with taxus and coronary artery bypass grafting) score (9 vs. 14 vs. 17.5; p<0.001) and duration of hospital stay (2 vs. 2 vs. 3; p<0.001) also increased with increasing SII_tertile_(tertile1 vs. tertile 2 vs. tertile 3). Systemic immune-inflammatory index was an independent predictor of SYNTAX score (ß: 0.232 [0.001 to 0.003]; p<0.001), extent of myocardial damage (ß: 0.152 [0 to 0.001]; p=0.005) and duration of hospital stay (ß: 0.168 [0.0 to 0.001]; p=0.003).

**Conclusions:**

This study has demonstrated that the systemic immune-inflammatory index, a simple hematological index, is a marker of atherosclerotic burden and longer hospital stay on well-known risk factors in high risk acute coronary syndrome patients.

## Introduction

Atherosclerosis is characterized by low grade chronic inflammation, which is interrupted by periods of acute exacerbations. These surges of inflammatory response accelerate the disease process and clinically manifest as acute coronary syndromes (ACS).^1 , 2^ ACS is common in patients with coronary artery disease (CAD), resulting in high mortality and morbidity rates.^3^ Accurate risk stratification in early disease course, therefore, is of paramount significance.

There are several inflammatory markers, such as C-reactive protein (CRP), tumor necrosis factor-α, and various interleukins, which are associated with poor outcome in the context of ACS.^4 , 5^ Simple hematological indices, such as neutrophil-lymphocyte ratio (NLR) and platelet-lymphocyte ratio (PLR) are also useful indicators of inflammation and promising prognostic factors in cardiovascular disease.^6 , 7^

In ACS, deranged activation of innate and adaptive immunity converges with platelet activation, resulting in thrombus formation. Systemic immune-inflammatory index (SII), which is derived from platelet, neutrophil and lymphocyte counts, combines all the main players of these pathophysiological pathways to represent the impaired balance. It was first described as a prognostic tool in hepatocellular carcinoma,^8^ which was followed by other solid tumors, such as those for colorectal, esophageal, and cervical cancers.^9^ SII was shown to predict survival better than other hematological indices, such as NLR or PLR in malignancies.^9^ Recently, Yang et al.^10^ have further demonstrated that SII predicts major cardiovascular events better than well-known cardiovascular risk factors in patients undergoing percutaneous coronary intervention (PCI).This study, therefore, aimed to explore the association of admission SII with atherosclerotic burden and early clinical outcomes in order to identify high-risk patients with ACS.

## Methods

After obtaining approval from the local ethics committee (2019/28/02/12), we retrospectively evaluated ACS patients who presented to the emergency department and were treated with coronary angiography between January 2018 and January 2019. There were 520 consecutive patients diagnosed with ACS — namely, unstable angina pectoris (UAP), non-ST elevation myocardial infarction (NSTEMI) and ST elevation myocardial infarction (STEMI) — based on their electrocardiographic, clinical and laboratory characteristics.^11 , 12^ Patients with high troponin concentrations due to a pathology other than ACS, with a known inflammatory/infectious disease, undergoing hemodialysis and with the diagnosis of myocardial infarction (MI) with normal coronary arteries, as well as those who did not undergo coronary angiography during the index hospitalization were not included in the study ( Figure 1 ). From the hospital records of 334 eligible patients, demographic characteristics, such as age, gender and presence of cardiovascular risk factors, such as hypertension, hyperlipidemia and diabetes mellitus (DM), were recorded. Patients with previous coronary artery bypass grafting (CABG) were not included into the analyses, as the association of SII with the atherosclerotic burden could not be stratified in this specific population. Laboratory parameters on admission to the emergency unit, such as hemoglobin, urea and creatinine concentration, as well as neutrophil, platelet and lymphocyte counts, were determined. Clinical outcomes to be evaluated were identified as atherosclerotic burden, extent of myocardial damage, occurrence of bleeding, acute kidney injury, duration of hospital stay and in-hospital mortality.

**Figure 1:** Flowchart for patient recruitment. CABG: coronary artery bypass grafting.

To determine the atherosclerotic burden, coronary angiograms were evaluated by two cardiologists, blinded to the study groups, who assessed the number of diseased vessels and the synergy between percutaneous coronary intervention (PCI) with taxus and CABG (SYNTAX) scores. Any epicardial coronary artery with 50% or more stenosis was identified as a diseased vessel. SYNTAX score was calculated as previously described.^13^ Maximum high sensitive troponin I (hs-cTnI) level represented the extent of myocardial damage. Bleeding was defined as a Hemoglobin (Hb) fall of 3 gr/dL or more during the hospital stay. Creatinine (Cr) levels during the hospital stay were obtained to calculate the increase from the baseline Cr level, in which an increase »0.3 mg/dL or 1.5 times baseline reflected the occurrence of acute kidney injury. SII was calculated as (platelet x neutrophil/lymphocyte), as described and studied previously.^8^ The study population was stratified into three groups with regard to SII levels.

### Statistical analysis

Statistical analyses were carried out using IBM^®^SPSS^®^Statistics for Mac, Version 20 software (IBM Corp., Armonk, New York). The continuous variables were presented as mean ±standard deviation (SD) or median (interquartile range); the categorical variables were presented as number and percentages. The variables were tested for normality of distribution by Kolmogorov-Smirnov Test. SII tertiles were compared by the one-way analysis of variance (ANOVA) in normally distributed variables and Kruskal-Wallis test in variables without normal distribution. Dunn’s test was used in the nonparametric pairwise comparisons if significant deviations were observed by Kruskal-Wallis test. Post hoc analysis in case of significant deviations showed by ANOVA was planned to be performed using Tukey’s or Tamhane’s test, depending on the homogeneity of variances. No post hoc analysis was performed for variables from which SII was driven. The categorical variables were compared by chi-squared. A p-value of 0.017 adjusted by the Bonferroni method was used in pairwise comparisons of categorical variables.

Correlation of hematological indices SII, NLR and PLR with SYNTAX score with respect to ACS type was tested with Spearman rank-order test. With setting each clinical outcome as the dependent variable, linear and logistic regression analyses were conducted, and the variables with a p value ≤0.2 in univariate comparisons were included into the multivariate model using the Stepwise method, in order to determine whether SII was a predictor of that specific clinical outcome. All necessary assumptions for the use of linear regression analysis were verified before results were interpreted. Results are presented with 95% confidence interval within [brackets]. Baseline variables such as age, gender, cardiovascular risk factors, creatinine, CRP and SII levels that are presented in Table 1 were included in the univariate analyses to determine possible predictors, but only the ones with a p value ≤0.2 were added to the multivariate model. A p value of <0.05 was considered statistically significant.

**Table 1:** Baseline characteristics and comparison of baseline characteristics in relation to systemic immune-inflammatory index tertiles

Variable	Total n=309	SII Tertile 1 n=103	SII Tertile 2 n=103	SII Tertile 3 n=103	1 vs. 2 p value	2 vs. 3 p value	1 vs. 3 p value
Age, years	64.5±12.1	62.8±11.8	65.1±13.1	65.5±11.2	0.500	1.00	0.306
Female gender, n (%)	82 (26.5)	25 (24.3)	32 (31.1)	25 (24.3)	0.350	0.350	1.00
UAP, n (%)	53 (17.2)	27 (26.2)	17 (16.5)	9 (8.7)	0.125	0.141	0.002
NSTEMI, n (%)	113 (36.6)	36 (35)	46 (44.7)	31 (30.1)	0.200	0.043	0.552
STEMI, n (%)	143 (46.3)	40 (38.8)	40 (38.8)	63 (61.2)	1.00	0.002	0.002
Diabetes mellitus, n (%)	123 (39.8)	36 (35)	40 (38.8)	47 (45.6)	0.665	0.397	0.155
Hypertension, n(%)	117 (37.9)	43 (41.7)	33 (32)	41 (39.8)	0.194	0.309	0.887
Hyperlipidemia, n (%)	125 (40.5)	42 (40.8)	36 (35)	47 (45.6)	0.473	0.155	0.574
Hemoglobin, g/dL	13.5±1	13.8±1.8	13.2±1.8	13.3±1.8	0.047	1.00	0.075
White blood cells, 103/µL	9.9±2.7	9.1±2.7	9.5±2.3	11.4±2.5	0.571	<0.001	<0.001
Neutrophil count, 103/µL	6.8 (3.54)	5.1 (2.37)	6.7 (2.5)	8.8 (3.03)	<0.001	<0.001	<0.001
Lymphocyte count, 103/µL	1.84 (1.1)	2.3 (1.12)	1.8 (0.89)	1.3 (0.75)	<0.001	<0.001	<0.001
Monocyte count, 103/µL	0.63 (0.36)	0.7 (0.36)	0.6 (0.31)	0.6 (0.39)	0.163	0.893	0.341
Platelet count, 103/µL	238 (96)	207 (75.75)	241 (75.00)	278 (116.75)	0.001	<0.001	<0.001
C-reactive protein, mg/L	4.55 (8.63)	3.8 (8.1)	4.7 (8.1)	5.4 (10.8)	0.651	0.262	0.271
Urea, mg/dL	34 (15)	32 (13)	33.5 (15)	36 (17.1)	0.469	0.082	0.038
Creatinine, mg/dL	0.93 (0.32)	0.93 (0.28)	0.91 (0.33)	0.94 (0.38)	0.984	0.694	0.854
SII	835 (860.09)	462 (194.51)	833 (252.9)	2055 (935.7)	<0.001	<0.001	<0.001

*p values represent the pairwise comparisons of the systemic immune-inflammatory index tertiles of the three patient groups without post hoc analysis . Note that the level of significance for the p value according to the Bonferroni correction is 0.017 in this table. UAP: unstable angina pectoris; NSTEMI: non-ST elevation myocardial infarction; STEMI: ST elevation myocardial infarction; SII: systemic immune-inflammatory index.

## Results

Baseline characteristics of the studied population and the comparison of these characteristics in relation to SII tertiles are presented in Table 1 . There was no difference among study groups in terms of cardiovascular risk factors such as age, gender, diabetes mellitus, hypertension and hyperlipidemia. When the SII groups were compared with regard to presenting ACS type, patients with UAP were more likely to be in the lowest SII tertile, while patients with STEMI were more likely to have higher SII. Patients with UAP were more likely to be in the lowest SII tertile, while patients with STEMI were more likely to have higher SII values ( Figure 2a ). To uncover the association of clinical presentation and SII, type of presenting ACS was evaluated within the SII spectrum, which showed that SII significantly differed with regard to type of ACS. Pairwise comparisons revealed that SII gradually increased from UAP to STEMI, in which the difference between UAP and STEMI was statistically significant ( Figure 2b ). The hemoglobin concentration, urea, creatinine and CRP levels were comparable within SII subgroups.

**Figure 2:** (a) Comparison of systemic immune-inflammatory index groups with regard to presenting acute coronary syndrome type; (b) Comparison of type of presenting acute coronary syndrome within the systemic immune-inflammatory index spectrum. UAP: unstable angina pectoris; NSTEMI: non-ST elevation myocardial infarction; STEMI: ST elevation myocardial infarction; ACS: acute coronary syndrome.

Table 2 summarizes the clinical outcomes in SII groups. The burden and complexity of coronary artery disease were reflected by the number of diseased vessels and the SYNTAX score of each patient, both of which demonstrated significant deflection among the SII groups. There was significantly lower number of diseased vessels in the lowest SII tertile when compared to other SII groups ( Figure 3a ). Moreover, the SYNTAX score increased remarkably as the SII tertile increased ( Figure 3b ). Maximal troponin level, which was the marker of myocardial damage, increased with increasing SII tertile, in which the differences between Tertile 1 vs. Tertile 3 and between Tertile 2 vs. Tertile 3 were statistically significant ( Figure 3c ). Hospital stay was longer in patients with the highest SII when compared to other two SII groups ( Figure 3d ). There was no significant difference among SII groups in terms of bleeding, acute kidney injury or in-hospital mortality.

**Table 2:** Clinical outcomes in relation to systemic immune-inflammatory index tertiles

Outcome	SII Tertile 1 n=103	SII Tertile 2 n=103	SII Tertile 3 n=103	1 vs 2 p value	2 vs 3 p value	1 vs 3 p value
Number of diseased vessels	1 (1)	2 (2)	2 (2)	0.013	1.00	0.011
Atherosclerotic burden or SYNTAX Score	9 (11)	14 (11.5)	17.5 (11)	0.022	0.002	<0.001
Extent of myocardial damage or Maximal troponin, ng/L	0.94 (2.06)	1.26 (3.61)	3 (4.02)	0.434	0.002	<0.001
Hospital stay, days	2 (1)	2 (1)	3 (3)	0.709	<0.001	0.026
Bleeding, n(%)	27 (26.2)	22 (21.4)	32 (31.1)	0.257*	0.157*	0.538*
Acute kidney injury, n(%)	18 (17.5)	17 (16.5)	22 (21.4)	1.00*	0.477*	0.598*
In-hospital mortality, n(%)	5 (4.9)	8 (7.8)	9 (8.7)	0.568*	1.00*	0.407*

*Note that the level of significance for the p values according to the Bonferroni correction is 0.017 for categorical variables. CABG: coronary artery bypass grafting; SII: systemic immune-inflammatory index.

**Figure 3:** Pairwise comparison of systemic immune-inflammatory index tertiles with regard to (a) Number of diseased vessels; (b) SYNTAX score; (c) Maximal troponin levels; (d) Hospital stay. SYNTAX: synergy between percutaneous coronary intervention with taxus and coronary artery bypass grafting.

Correlations of hematological indices SII, NLR and PLR with SYNTAX score with respect to ACS type were evaluated ( Table 3 ). In UAP patients, NLR and PLR were not correlated with SYNTAX score, while there was a positive correlation between SII and SYNTAX score. In NSTEMI patients, SII, NLR and PLR were positively correlated with SYNTAX score. Similarly, all three indices were positively correlated with SYNTAX score in STEMI patients.

**Table 3:** Correlation of SYNTAX score with systemic immune-inflammatory index, neutrophil-lymphocyte ratio and platelet-lymphocyte ratio with regard to type of presenting acute coronary syndrome.

		SII			NLR		PLR	
		r_s_	p		r_s_	p	r_s_	p
UAP	SYNTAX	0.300	0.031		0.266	0.054	0.145	0.299
NSTEMI	SYNTAX	0.345	<0.001		0.236	0.011	0.183	0.045
STEMI	SYNTAX	0.471		<0.001	0.456	<0.001	0.387	<0.001

*SII: systemic immune-inflammatory index; NLR: neutrophile-lymphocyte ratio; PLR: platelet-lymphocyte ratio; UAP: unstable angina pectoris; NSTEMI: non-ST elevation myocardial infarction; STEMI: ST elevation myocardial infarction.*

Clinical outcomes tested by multivariate linear regression analyses were atherosclerotic burden represented by SYNTAX score, extent of myocardial damage (maximal troponin concentration) and duration of hospital stay. Linear regression revealed that SII (ß: 0.232 [0.001 to 0.003]; p<0.001), age (ß: 0.156 [0.019 to 0.165]; p=0.014) and DM (ß: 0.165 [0.935 to 4.42]; p=0.003) were significant predictors of SYNTAX score. The independent predictors of the extent of myocardial damage were SII (ß: 0.152 [0 to 0.001]; p=0.005); female gender (ß: -0.147 [-1.801 to -0.271]; p=0.008) and DM (ß: 0.142 [0.197 to 1.557]; p=0.012). Similarly, the independent predictors of duration of hospital stay were SII (ß: 0.168 [0.0 to 0.001]; p=0.003) and presence of diabetes mellitus (ß: 0.124 [0.095 to 1.74]; p=0.029).

Clinical outcomes tested by logistic regression analyses were bleeding and in-hospital mortality. Binary logistic regression indicated that an independent predictor of bleeding was baseline hemoglobin value (odds ratio (OR): 1.29 [1.09 to 1.52] p=0.002). For in-hospital mortality, only age (OR: 1.09 [1.035 to 1.155] p=0.001) and baseline creatinine (OR: 4.6 [1.137 to 18.787] p=0.032) were predictors. SII was not a predictor in neither of outcomes.

## Discussion

In this retrospective cohort of ACS patients, we demonstrated that a simple hematological index derived from neutrophil, lymphocyte and platelet counts on admission can be utilized to extrapolate the atherosclerotic burden, the extent of myocardial damage and the duration of hospital stay, independent of traditional cardiovascular risk factors and inflammatory markers such as CRP. Correlation of SII with SYNTAX score persisted in ACS patients irrespective of presence or absence of necrosis. This finding suggests that SII can potentially be used to identify high-risk individuals in ACS as early as on admission.

Mortality and morbidity in cardiovascular events are multifactorial, and results from a confluence of differing pathophysiological pathways in which inflammation plays a central role.^1^ Although severe systemic inflammation is an established indicator of mortality in ACS, no single inflammatory biomarker could have been identified to guide the treatment of cardiovascular risk.^14^ Even CRP, whose role in inflammation and atherosclerosis is well established, can only modestly predict cardiovascular events.^15^ Several hematological indices, such as NLR or PLR, have been proposed to represent early inflammatory response in ACS and possess prognostic significance.^16^ Nevertheless, inflammation is a continuous process and evidence revealed that a plethora of circulating cytokines subsides after an acute event with persisting high concentrations of some cytokines, such as interleukin-6 in the early post-MI period.^17^

Of white blood cell subtypes, neutrophils are the key elements of nonspecific first line defense. Neutrophil count is a well-defined prognostic factor in cardiovascular disease, particularly in patients with ACS.^18 , 19^ Lymphocytes, on the other hand, are part of adaptive immunity, which alleviates inflammation through B_1_, T-helper_2_and T-regulatory subtypes.^1 , 20^ Lymphocyte count decreases secondary to acute stress hormones following myocardial infarction.^21^ Clinical studies have also established the association of low lymphocyte count with increased in-hospital mortality.^21^ A high NLR, thus, is associated with worse clinical outcomes, both in patients with ACS and in those with stable CAD undergoing percutaneous coronary intervention.^6 , 7^ NLR was also shown to be associated with severity and complexity of CAD, as represented by SYNTAX score.^6^

On the basis of evidence that shows close leukocyte-platelet interaction in inflammation triggering thrombosis, Choi et al.^22^ have combined NLR with mean platelet volume and showed that addition of a platelet related index predicts future cardiac events better, especially in patients with ACS. Çiçek et al.^7^ have also shown that a combination of NLR and PLR increased the power of predicting poor short and long-term prognosis when compared to using them alone in patients undergoing primary PCI. In this study, we speculated that an index that combines NLR and PLR would better represent the inflammo-thrombotic status of patients in the peri-MI period.

There is scarce data on the value of SII in cardiovascular diseases. Yang et al.^10^ have evaluated the role of SII in patients undergoing PCI and showed that, on a follow-up of 54.6±35.1 months, SII predicted major cardiac events such as cardiovascular death, nonfatal MI and nonfatal stroke better than traditional cardiovascular risk factors. They simply suggested that inflammation quantified by SII accounted for the poor clinical outcomes. They did not, though, report the association of SII with complexity of CAD. For the first time in literature, we have demonstrated that the SII was independently associated with SYNTAX score, irrespective of CRP and other well-known cardiovascular risk factors. Our finding also explains the results of Yang et al.,^10^ since patients with higher SII and SYNTAX scores are more prone to future cardiovascular events.

Herein, it was intriguing to find a positive correlation of SII with SYNTAX score in all ACS subtypes. Among three hematological indices, namely NLR, PLR and SII, only SII was correlated with SYNTAX score in UAP patients without severe myocardial necrosis. ACS studies showing the relation of NLR and PLR with atherosclerotic burden were mostly derived from patients with either non-ST MI or STEMI with severe myocardial necrosis and significantly elevated troponin concentrations.^23 , 24^ Moreover, SII was profoundly influenced by the type of the presenting ACS; in which STEMI patients had remarkably higher SII indices and UAP patients had lower SII indices. These findings suggest that, contrary to simple perception that considers SII as the result of inflammatory surge around the time of ACS, SII reflects the intertwined baseline atherosclerotic process prone to complications.

SII was related to the extent of myocardial damage and this relationship was probably through the type of ACS, with higher SII values in STEMI patients. Our finding is in line with the previous studies which have shown that, in the absence of necrosis, correlation of white blood cell count (WBC) with mortality was diminished.^25 , 26^ Nunez et al.^26^ have found weaker association of WBC with non-STEMI when compared to STEMI, and speculated that the greater the extent of necrosis, the larger the WBC response. Our results suggest that SII, which integrates platelet count to the WBC counts, can define residual ongoing inflammation better than other hematological indices, even in the absence of necrosis.

In this study, SII was a predictor of duration of hospital stay in patients with ACS. Increasing SII values represent high-risk patients with high SYNTAX score and severe myocardial damage, which explains the reason of longer durations of hospital care. Herein, we did not demonstrate an association between SII and in-hospital mortality, which can be attributed to early treatment with primary percutaneous intervention and relatively short duration of hospital stay.

### Study limitations

This study has several limitations regarding the retrospective nature of the study design and lack of follow-up data. Patient selection was elaborate as we have tried to exclude all patients with active infection or inflammatory condition, thus decreasing the number of subjects. Additionally, we did not include patients with CABG, since atherosclerotic burden could not be determined by neither number of diseased vessels nor SYNTAX score in patients with previous CABG. Another limitation of the current study was the fact that we could not include the baseline medication of the study population into the analyses.

## Conclusions

Immune, inflammatory and thrombotic balance is of pivotal importance in the pathogenesis of ACS. Given the distinctive association of SII with SYNTAX score, extent of myocardial damage and duration of hospital stay, it can potentially be used to identify high risk patients through a readily available and inexpensive approach.
